# Identification of predictors of bone mineral density trajectories in pediatric Systemic Lupus Erythematosus patients

**DOI:** 10.1186/1546-0096-9-S1-P262

**Published:** 2011-09-14

**Authors:** SHL Lim, SM Benseler, PN Tyrrell, M Charron, ED Silverman

**Affiliations:** 1 Division of Rheumatology, Toronto, Canada; 2 Division of Nuclear Medicine, Toronto, Canada

## Background

Children with pediatric Systemic Lupus Erythematosus (pSLE) are at risk of low bone mass. No previous study had focused on longitudinal changes in bone mineral density (BMD) among children with pSLE from diagnosis.

## Aims

1) To identify the trajectories of lumbar spine (LS) BMD in pSLE patients and 2) To identify predictors of this change in BMD over time.

## Methods

All 68 newly diagnosed patients with pSLE prospectively followed in our Lupus clinic cohort who had 3 serial Dual Energy X-ray Absorptiometry (DEXA) examinations were studied. Low lumbar spine (LS) BMD was defined as z-score ≤ -2.0. Trajectory of LS BMD change and predictors of this change were assessed by Hierarchical Linear Modeling (HLM).

## Results

Females constituted 84% of the cohort with a median age at diagnosis of 13.1 years. The mean LS BMD z-scores decreased with time. Initially, 9% of patients had a low BMD, this proportion increased to 19% by 3 years after diagnosis. Overall, the BMD category decreased in 35% of patients from 1^st^ to 3rd DEXA study. LS BMD followed a general deteriorating trajectory of -0.25 z-score/year from diagnosis. Body mass index (BMI) z-score and cumulative steroid dose were the best predictors of BMD trajectories over time.

## Conclusions

The LS BMD of pSLE patients decreased at a rate of 0.25 z-score/year. BMI z-score and cumulative doses of steroids modified the trajectories of LS BMD. These factors are potentially modifiable targets that can improve individual patient’s BMD trajectory.

